# Inflammatory Chemokine Expression via Toll-Like Receptor 3 Signaling in Normal Human Mesangial Cells

**DOI:** 10.1155/2013/984708

**Published:** 2013-06-26

**Authors:** Hiroshi Tanaka, Tadaatsu Imaizumi

**Affiliations:** ^1^Department of School Health Science, Hirosaki University Faculty of Education, 1 Bunkyo-cho, Hirosaki 036-8560, Japan; ^2^Department of Pediatrics, Hirosaki University Hospital, Hirosaki 036-8563, Japan; ^3^Department of Vascular Biology, Graduate School of Medicine, Hirosaki University, Hirosaki 036-8562, Japan

## Abstract

The innate and adaptive immune systems have been reported to play an important role in the pathogenesis of glomerular diseases. Since viral infections may trigger the development of inflammatory renal disease or the worsening of preexisting renal disease, recent studies have focused on the involvement of toll-like receptors (TLRs) and their signaling pathways in the inflammatory processes of glomerular cells. Viral double-stranded RNA (dsRNA) can activate not only TLR3 located within intracellular endosomes but also retinoic-acid-inducible-gene-I- (RIG-I-) like helicase receptors located within the cytosol. RIG-I and melanoma differentiation-associated gene 5 (MDA5) are members of the RNA helicase family in the cytosol, and both act as pathogen recognition receptors. The activation of TLRs and their downstream immune responses can be induced by both infectious pathogens and noninfectious stimuli such as endogenous ligands, and this mechanism may be involved in the pathogenesis of autoimmune renal diseases. However, there are few data on the interaction between TLR3, MDA5, and RIG-I in autoimmune glomerular diseases. Based on our recent experimental studies using cultured normal human mesangial cells (MCs), we found that novel TLR3-mediated signaling pathways in MCs may be involved in the pathogenesis of glomerular diseases. In the present paper, we summarize our recent findings.

## 1. Introduction

The innate and adaptive immune systems have been reported to play an important role in the pathogenesis of glomerular diseases. Since viral infections may trigger the development of inflammatory renal disease or the worsening of preexisting renal disease, recent studies have focused on the involvement of toll-like receptors (TLRs) and their signaling pathways in the inflammatory processes of glomerular cells [1]. The activation of TLRs and their downstream immune responses can be induced by both infectious pathogens and noninfectious stimuli such as endogenous ligands, and this mechanism may be possibly involved in the pathogenesis of autoimmune renal diseases [1–3]. Viral double-stranded RNA (dsRNA) can activate both TLR3 located within intracellular endosomes and retinoic acid-inducible gene-I (RIG-I)-like helicase receptors located within the cytosol [4]. RIG-I and melanoma differentiation-associated gene 5 (MDA5) are members of the RNA helicase family in the cytosol, and both act as pathogen recognition receptors [5]. Therefore, RIG-I and MDA5 may also be involved in the pathogenesis of autoimmune renal diseases [6–14]. Recent studies have revealed the expressions of TLRs in resident renal cells, suggesting the involvement of the expression of TLRs and activation of their downstream signaling pathway in the pathogenesis of glomerular diseases [1–3]. Once presumptive antigenic ligands bind to TLRs, the activation of transcriptional factors, such as interferon regulatory factors (IRFs) and nuclear factor kappa B (NF-*κ*B) is induced through intracellular signaling cascade activation. This activation results in the release of adhesion molecules, cytokines, and chemokines, which play a pivotal role in innate and adaptive immune responses [1–3]. 

Since glomerular mesangial cells (MCs) have been reported to produce a wide variety of proinflammatory molecules that play an important role in immune and inflammatory reactions in the kidney [15], MCs itself are now thought to play a pivotal role in the pathogenesis of renal diseases [16]. Indeed, the activation of mesangial TLR3 induced by polyinosinic-polycytidylic acid (poly IC), a synthetic analogue of viral dsRNA, upregulated the expression of functional molecules such as interleukin (IL)-6 [15], CC chemokine ligand (CCL) 2 (or monocyte-chemoattractant protein-1) [6], CCL5 (or regulated on activation, normal T-cell expression and secretion) [17], matrix metalloproteinase 9, plasminogen activator inhibitor type 1, and tissue plasminogen activator in human MCs. These findings suggest that viral RNA can influence, at least in part, the generation and degradation of the extracellular matrix in the mesangium in ways other than through direct viral stimulation and that glomerulosclerosis might subsequently develop [8, 18], although this theory remains speculative. However, the precise role of the interaction between TLR3, MDA5, and RIG-I in mesangial inflammation in human glomerular diseases remains to be elucidated. 

In our previous studies, we had observed high levels of RIG-I expression in the glomeruli and urinary sediments of patients with lupus nephritis [7, 19]. RIG-I overexpression may be a pathological feature of lupus nephritis. Further, we recently observed intense glomerular expression of human myxovirus resistance protein 1, a type-I-interferon- (IFN-) dependent transcript, in biopsy specimens from patients with lupus nephritis, whereas negative staining occurred in specimens from patients with IgA nephropathy or purpura nephritis. Interestingly, it has been reported that the implication of “psuedoviral” immunity as a novel disease concept of lupus nephritis, that is, self-nucleic acid particles resembling viral particles activates viral nucleic sensors and subsequent type I IFNs production [20]. The nucleic acid-specific TLR3, TLR7, TLR8, and TLR9 cover a spectrum of different endogenous ligands as well as viral RNA formats, and the activation of these TLRs signaling cascades result in inducing type I IFNs release [20]. Also, increased type I IFNs produced by resident renal cells have been associated with lupus nephritis, that is, type I IFNs directory promote and modulate pathogenesis of murine lupus nephritis [21]. Thus, recent studies support, at least in part, the theory of innate immune system activation in the pathogenesis of lupus nephritis. To examine this theory, we recently performed several experiments using cultured normal human MCs treated with poly IC and/or a poly IC/cationic lipid complex and found the involvement of novel TLR3-mediated signaling pathways that upregulate inflammatory chemokine expression during mesangial inflammation in human MCs [10–14, 22]. Poly IC has been widely used to mimic viral infection in various cell types including MCs. Accordingly, we used poly IC in our recent experiments. The treatment with poly IC is a model of cells exposed to viral dsRNA released from dying cells, whereas treatment with poly IC/cationic lipid complex is a model of cytosolic viral dsRNA recognition [13, 22]. 

In the present paper, we summarize our recent findings of mesangial inflammation via TLR3-mediated signaling pathways. Details of our experimental procedures and results obtained were published previously in our recent papers [10–14, 22]. We believe that these novel pathways may be involved in the pathogenesis of human autoimmune glomerular diseases.

## 2. TLR3 and RIG-I in Human MCs

**Figure 1:**
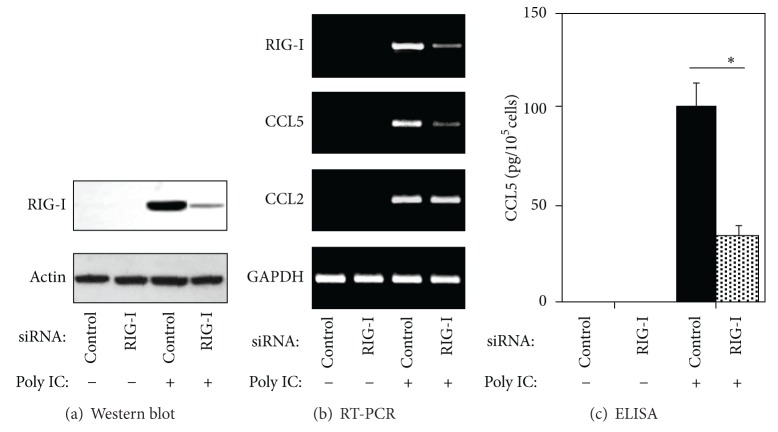
Knockdown of RIG-I reduces the poly IC-induced expression of CCL5 in human MCs. The cells were transfected with siRNA against RIG-I or control siRNA and then stimulated with 20 *μ*g/mL of poly IC (Imaizumi et al. NDT 2010). (a) After 24 h of poly IC treatment, the cells were lysed, and western blotting for CCL5 was performed. (b) The cells were incubated for 16 h with poly IC, RNA was extracted, and RT-PCR was performed for RIG-I, CCL5, and CCL2. (c) The culture medium was collected after 24 h, and the concentration of CCL5 was determined by ELISA (*n* = 3, *P < 0.01).

To evaluate the potential role of RIG-I in response to viral dsRNA in human MCs, we treated the cells with poly IC, an authentic viral dsRNA. In this experiment, the cells were simply treated with poly IC and not transfected using the poly/cationic lipid complex. Stimulation with poly IC resulted in increased expression of both RIG-I mRNA and protein in a concentration-dependent and time-dependent manner accompanied by CCL5 expression [11]. Furthermore, treatment with RIG-I small interfering RNAs (siRNA) significantly lowered poly IC-induced CCL5 expression. In contrast, the poly IC-induced expression of CCL2 mRNA was not affected by RIG-I siRNA (Figure 1). Interestingly, the poly IC-induced RIG-I expression was suppressed in response to treatment with siRNA against TLR3. In addition, TLR3 siRNA downregulated the poly IC-induced expressions of TLR3 and interferon (IFN)-*β*, whereas RIG-I siRNA did not affect the expression of either TLR3 or IFN-*β*. Interestingly, poly IC treatment did not induce IFN-*α* or IFN-*γ* in this experiment [11]. Thus, IFN-*β* siRNA was used to examine the role of IFN-*β* as a potential mediator of poly IC-induced RIG-I expression. As a result, the poly IC-induced expressions of IFN-*β* and RIG-I were markedly inhibited in cells transfected with IFN-*β* siRNA. Pretreatment of the cells with a blocking antibody against the type I IFN receptor also reduced the poly IC-induced expression of RIG-I. On the contrary, the expression of both RIG-I and CCL5 was induced after transfection of the cells with IFN-*β* expression plasmid [11]. Moreover, pretreatment of the cells with dexamethasone reduced the poly IC-induced expression of both RIG-I and IFN-*β*, whereas this treatment had no effect on IFN-*β*-induced RIG-I expression [11]. 

Our results suggested that the expression of CCL5 was selectively regulated by RIG-I expression in human MCs because poly IC-induced CCL5 expression was inhibited in response to RIG-I knockdown, whereas CCL2 expression was not affected by RIG-I siRNA treatment. It has been reported that viral dsRNA activates human and murine MCs to produce IL-6 and CCL2 via TLR3 [6, 17]. Further, viral nucleic acids occur in formats other than dsRNA, such as 5′-triphosphate RNA (3P-RNA) can activate murine MCs via TLR3-independent RIG-I pathways, suggesting complexed 3P-RNA and dsRNA trigger antiviral responses via both TLR3-dependent and independent pathways in MCs, which may promote glomerulonephritis, although the role of RIG-I in innate pathogen recognition can vary between cell types and species [23]. A recent report suggested that RIG-I, but not TLR3, mediated the secretion of type I IFN in poly IC/cationic lipid complex-treated cultured murine glomerular endothelial cells [9]. The cross talk between glomerular endothelial cells and MCs may be an important aspect of glomerular inflammation, and the RIG-I/CCL5 pathway in MCs may contribute to glomerular inflammation, although the implication of RIG-I may vary between species. Our recent study showed that RIG-I may function downstream to TLR3 in the signaling cascade activated by poly IC-induced expression of CCL5 in human MCs [11]. In addition, the inhibitory effect of dexamethasone against CCL5 expression may depend on the suppression of IFN-*β* production, but not on the IFN-*β*-induced RIG-I expression. In this signaling pathway in MCs, TLR3 and newly synthesized IFN-*β* are involved in poly IC-induced RIG-I expression. Since dexamethasone had no effect on IFN-*β*-induced RIG-I expression, the inhibitory effect of dexamethasone may depend on the suppression of IFN-*β* production [10–12]. 

## 3. TLR3, MDA5, and RIG-I in MCs

MDA5 and RIG-I were recently shown to function as pathogen recognition receptors of viral dsRNA in the cytostome, and both receptors may play an important role in innate immune reactions [4, 5]. Although the expression of MDA has been documented in human MCs [13] as well as murine MCs [15], the detailed implications of MDA5 expression in human MCs have not yet been clarified. Since C-X-C motif chemokine 10 (CXCL10, also known as IFN-*γ*-induced protein 10), a chemokine with chemotactic activity for leukocytes with CXCR3, is thought to be involved in the pathogenesis of glomerular diseases [23], we examined the effect of poly IC and the role of MDA5 in CXCL10 expression in cultured human MCs [13, 14]. Poly IC, either simply applied to the cells or transfected as a complex with a cationic lipid, induced MDA5 expression in a concentration-dependent and time-dependent manner. TLR3, localized in the endosomes, is thought to serve as a receptor for nontransfected poly IC, whereas RIG-I and MDA5, localized in the cytoplasm, are thought to serve as receptors for transfected poly IC in this experiment. Transfection of the cells with siRNA against TLR3 suppressed the poly IC-induced expression of MDA5 mRNA and protein, while siRNA against TLR3 did not suppress poly IC/cationic lipid complex-induced MDA5 expression. On the other hand, the siRNA against RIG-I clearly inhibited the MDA5 expression induced by the poly IC/cationic lipid complex, whereas MDA5 knockdown had no effect on RIG-I expression induced by poly IC or the poly IC/cationic lipid complex. Thus, MDA5 may be located downstream of RIG-I in this signaling pathway in cultured human MCs [13]. Interestingly, these results are inconsistent with those of an earlier report of MDA5 expression in murine MCs [15]. The molecular mechanisms of pathogen recognition may vary among species [23], but this issue remains to be elucidated in future studies [12]. Since we observed that IFN-*β*, not IFN-*α*, is a key mediator of MDA5 expression in MCs, as suggested by our recent examinations of poly IC-induced TLR3 signaling pathways in MCs [11, 12], induction of IFN-*β* mRNA was confirmed in the cells treated with poly IC as well as those transfected with the poly IC/cationic lipid complex. In the present experiment, TLR3 knockdown suppressed IFN-*β* induction in the poly IC-treated cells, while RIG-I knockdown suppressed that induction in the cells transfected with poly IC/cationic lipid. Transfection of the cells with IFN-*β* siRNA markedly inhibited production of MDA5 and CXCL10 induced by poly IC treatment or poly IC/cationic lipid transfection. On the other hand, MDA5 was markedly induced by transfection with an IFN-*β* expression plasmid. This finding suggests that newly synthesized IFN-*β* mediates poly IC-induced MDA5 expression. In the present study, we observed that IFN-*β* is induced either by poly IC or a poly IC/cationic lipid complex and that *de novo* synthesized IFN-*β* may mediate the expression of MDA5 [13]. Apart from its antiviral property, IFN-*β* is thought to be an important mediator in virus-associated glomerulonephritis and immune complex-mediated glomerulonephritis exacerbated by viral infections [24]. Interestingly, RIG-I was involved in IFN-*β* expression induced by the poly IC/cationic lipid complex, but not in the MDA5 expression induced by IFN-*β*. Expression of CXCL10 in resting cells was faint and was markedly upregulated by treatment of cells with poly IC or by transfection of cells with poly IC/cationic lipid complex transfection. Knockdown of MDA5 resulted in partial inhibition of CXCL10 induction by poly IC or poly IC/cationic lipid complex [13]. Taking together, the involvement of the TLR3/IFN-*β*/MDA5/CXCL10 and the RIG-I/IFN-*β*/MDA5/CXCL10 pathways and possible interaction between these signaling cascades may play an important role in immune and inflammatory reactions against both viral and “pseudoviral” infections [20] in human MCs, although these observations remain preliminary.

**Figure 2:**
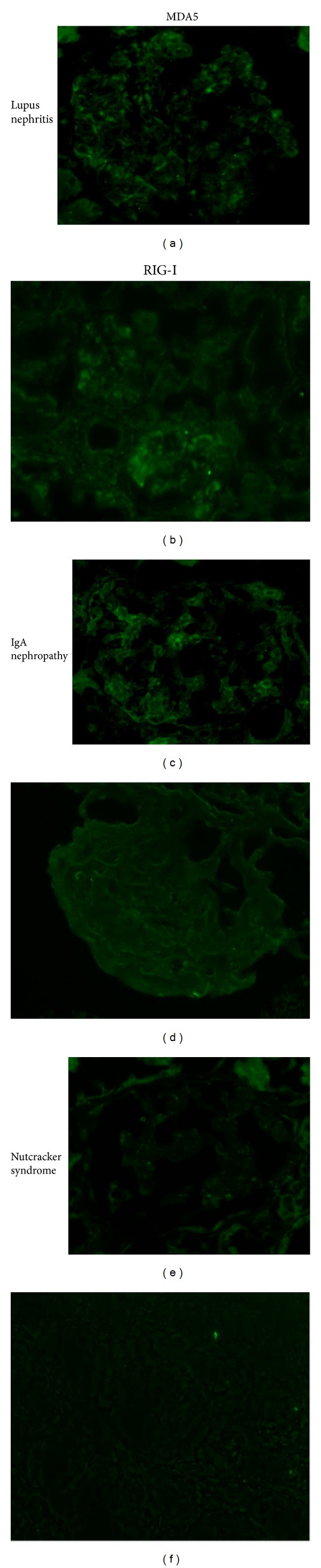
Immunoreactivity of MDA5 and RIG-I in biopsy specimens (Imaizumi et al. Tohoku J Exp Med 2012). Stored kidney specimens in good condition obtained from cases of diffuse proliferative lupus nephritis, proteinuric IgA nephropathy, minimal change nephrotic syndrome, and nutcracker syndrome were used for immunofluorescent study of MDA5 and RIG-I expression. After blocking by incubation with 1% goat serum, the slides were incubated with an anti-MDA5 antibody (1 : 100) or an anti-RIG-I antibody (1 : 1000). Intense MDA5 immunoreactivity was detected in MCs of the specimens from diffuse proliferative lupus nephritis and proteinuric IgA nephropathy, while the expression in nonimmune complex-mediated renal diseases was undetectable. Interestingly, RIG-I immunoreactivity was only in diffuse proliferative lupus nephritis.

Further, we observed mesangial MDA5 immunoreactivity in biopsy specimens from patients with severe lupus nephritis and proteinuric IgA nephropathy (urinary protein excretion/urinary creatinine >1.0) but no MDA5 expression in patients with noninflammatory renal diseases (Figure 2). Interestingly, there was no mesangial expression of RIG-I in the specimens from patients with IgA nephropathy despite the positive MDA5 staining. These observations suggested that the expression of MDA5 in severe lupus nephritis is associated with signaling pathway activation via RIG-I [7], whereas MDA5 expression in IgA nephropathy may be RIG-I-independent. The differential roles of MDA5 and RIG-I in severe lupus nephritis and proteinuric IgA nephropathy may predict the specific molecular mechanisms of these glomerulonephritis forms. In this context, we previously observed that mesangial expression of RIG-I was induced by IFN-*γ*, and which may promote inflammatory process in the pathogenesis of lupus nephritis [10]. This issue should be further investigated in future studies.

## 4. Interaction between Interferon-Stimulated Gene (ISG) 56, MDA5, and RIG-I

It has been reported that the IFN-stimulated gene 56 (ISG56) regulates cellular function and can be induced by IFN, dsRNA, or viruses in most cell types [25]. Since ISG56 expression in mouse MCs has been reported [26], we recently examined whether ISG 56 expression is involved in TLR3 signaling, which induces CCL5 [11] and CXCL10 [13] in human MCs. When the cells were treated with poly IC, ISG56 mRNA and protein were markedly increased in a concentration-dependent and time-dependent manner. The induction of ISG56 mRNA and protein by this treatment was inhibited by siRNA against TLR3 or IFN-*β*. On the contrary, overexpression of IFN-*β* by IFN-*β* plasmid transfection resulted in significant induction of ISG56. The transfection of cells with siRNA against ISG56 had no effect on IFN-*β* expression but significantly decreased the expressions of MDA5, RIG-I, CXCL10, and CCL5 mRNA and protein [14]. Knockdown of ISG56 did not affect cell viability. Since IFN-*β*, but neither IFN-*α* nor IFN-*γ*, was induced by poly IC treatment in MCs under the conditions used here [11, 12], we conclude that IFN-*β* is a major player in the TLR3 signaling within MCs [11–14]. As a result, we confirmed that newly synthesized IFN-*β* is involved in poly IC-induced ISG56 expression [14]. 

**Figure 3:**
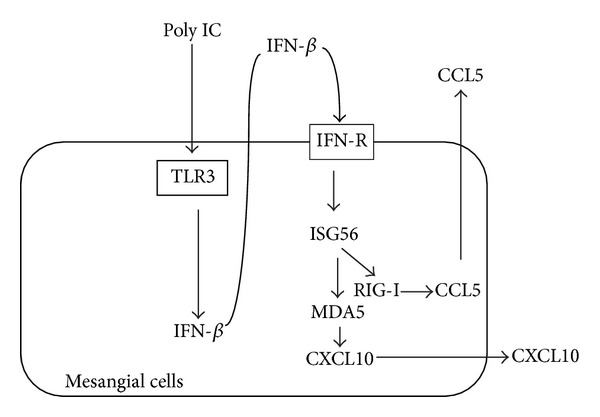
Proposed RIG-I- and MDA5-mediated signaling pathways via TLR3 activation induced by poly IC in human mesangial cells. (NDT 2010, Tohoku J Exp Med 2012, Am J Nephrol 2013).

We previously showed that TLR3 signaling in human MCs induces CCL5 and CXCL10 via the TLR3/IFN-*β*/RIG-I/CCL5 [11] and TLR3/IFN-*β*/MDA5/CXCL10 [13] axes, respectively. Further, this study suggests that ISG56 regulates the expression of MDA5/CXCL10 and RIG-I/CCL5 pathways in the downstream of TLR3/IFN-*β* [14]. Increased expression of RIG-I [7, 13] and MDA5 [13] has been observed in renal biopsy specimens from patients with proliferative lupus nephritis, while only MDA immunoreactivity was observed in biopsy specimens from patients with proteinuric IgA nephropathy. The differential roles of RIG-I and MDA5 in proliferative lupus nephritis and proteinuric IgA nephropathy may predict specific molecular mechanisms for these diseases [13]. Taking together, ISG56 may also be involved in inflammatory renal diseases, although this theory remains speculative. Further detailed studies are needed to resolve this issue. Proposed inflammatory pathways via TLR3 signaling in MCs are shown in Figure 3.

## 5. Conclusion

We believe that involvement of the novel TLR3/IFN-*β*/RIG-I/CCL5 and TLR3/IFN-*β*/MDA5/CXCL10 signaling pathways in MCs may contribute to mesangial inflammation. Cross talk between these signaling pathways may be involved in the pathogenesis of human glomerulonephritis including lupus nephritis and in the aggravation of glomerulonephritis due to both viral and “pseudoviral” infections. Since the inhibitory effect of dexamethasone may depend on the suppression of IFN-*β* production and not on IFN-*β*-induced RIG-I and MDA5 expressions [10–12], effective treatment strategies for the intervening in these signaling pathways are needed, although this remains to be elucidated in future studies. We believe that intervention within these signaling pathways may lead to the development of future therapeutic strategies for glomerular diseases. 

We found the involvement of novel RIG-I-mediated and MDA5-mediated signaling pathways in mesangial inflammation in human MCs that differed from TLR3 triggering, which demonstrated the clinical significance of this issue.
